# Cellular Angiofibroma: A Rare Vulvar Tumor Case Report

**DOI:** 10.1055/s-0040-1712485

**Published:** 2020-06

**Authors:** Ana Helena Barbosa Fachada, Cátia Sofia Guilherme Ferreira Pais, Marta Andrea Ferreira Fernandes, Nuno Jorge Lopes Dias, António Manuel Leitão Loureiro Pipa

**Affiliations:** 1 Gynecology and Obstetrics Department, Centro Hospitalar Tondela-Viseu, Viseu, Portugal; 2 Anatomic Pathology Department, Centro Hospitalar Tondela-Viseu, Viseu, Portugal

**Keywords:** cellular angiofibroma, mesenchymal tumor, vulva, angiofibroma celular, tumor mesenquimatoso, vulva

## Abstract

Cellular angiofibroma (CA)is a rare benign mesenchymal tumor. In women, it occurs mainly in the vulvovaginal region, with vulvar location in 70% of the cases. Its clinical presentation is nonspecific and similar to several other vulvar tumors of different cellular origins. Thus, its histological and immunohistochemical features allow distinction from other tumors. Cellular angiofibromas have good prognosis, despite some risk of relapse. The authors present the case of a 49-year-old woman with a bulky right vulvar lesion, for which the preoperative diagnosis was a Bartholin cyst, but the histological and immunohistochemical evaluation yielded a CA.

## Resumo

O angiofibroma celular é um tumor mesenquimatoso benigno e raro. Nas mulheres, surge principalmente na região vulvo-vaginal, com localização vulvar em 70% dos casos. A sua apresentação clínica é inespecífica e semelhante a vários outros tumores vulvares de diferentes origens celulares. Assim, são as suas características histológicas e imunohistoquímicas que permitem a diferenciação entre eles. Os angiofibromas celulares têm bom prognóstico, embora apresentem algum risco de recidiva. Os autores apresentam o caso de uma mulher de 49 anos de idade com uma lesão vulvar direita volumosa, cujo diagnóstico pré-operatório era de quisto da glândula de Bartholin, mas cujo exame histológico e imunohistoquímico revelaram tratar-se de um angiofibroma celular.

## Introduction

Cellular angiofibroma (CA) is a rare benign tumor, first described in 1997 by Nucci et al.^1^ It is derived from the mesenchymal cells of the blood vessels in superficial soft tissue, and it occurs predominantly in the distal genital tract of both genders: vulvovaginal region in women and inguinoscrotal region in men.^2^ ^3^ ^4^ The CA does not show morphologic differences between the two genders^2^ ^3^ ^4^ and, in women, it grows especially in the vulva, so that 70% of cases are in these region.^2^

Cellular angiofibroma lesions are characterized by occurring in middle-aged women (fifth decade of life) but later in men (seventh decade of life).^2^ ^3^ Usually, CAs are small sized (< 3 cm), painless, and well-circumscribed tumors. They have a slow growth, and, although benign, there are a few cases of focal invasion and a case of tumor recurrence reported in the literature.^4^ ^5^ Still, they have not yet been associated with aggressive clinical behavior.^4^ ^5^ ^6^ ^7^

## Case Report

A 49-year-old woman was referred to gynecological evaluation at Centro Hospitalar Tondela-Viseu, in Portugal, for a right vulvar lesion that had first been observed 2 years earlier. She reported a fast enlargement in the months prior to the appointment and no previous history of pain, bleeding, or infection.

The patient's menarche was at 15 years old, she had 5 pregnancies with 5 eutocic births and was submitted to bilateral tubal ligation at 32 years of age. She denied any personal or family history of breast or gynecological cancer.

Gynecological examination revealed a soft, mobile, and painless vulvar mass involving the right labium majus with ∼ 5 cm in size, and no other findings in the pelvic physical exam. Clinical diagnosis was a Bartholin gland cyst, and a simple excision surgery was performed in the operation room , with no surgical complications.

**Fig. 1:**
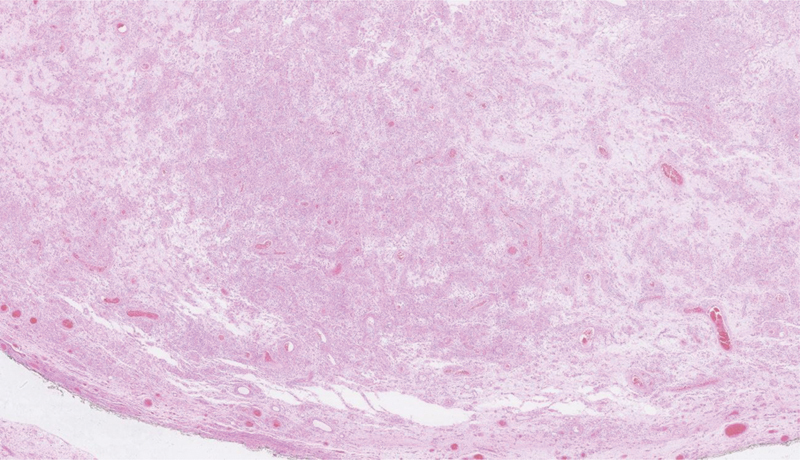
Tumor with sharp boundary and regular contour, with various cellular and edematous/myxoid areas.

**Fig. 2:**
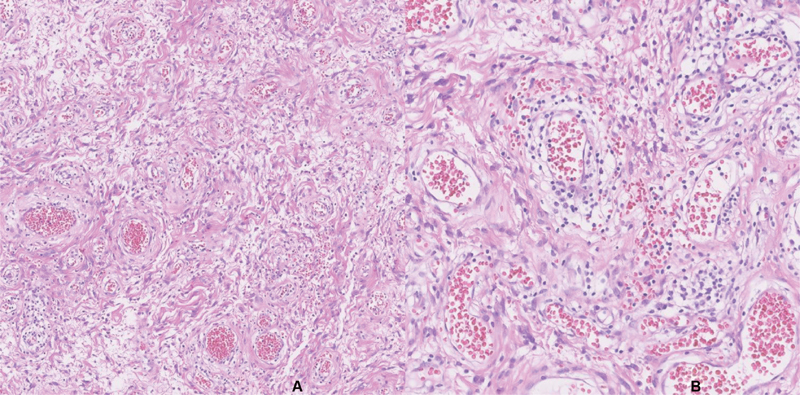
Multiple blood vessels of variably thick wall, surrounded by edematous matrix with collagen fibers (**A**), where spindle cells are promptly seen, without significant atypia or mitoses (**B**).

**Fig. 3:**
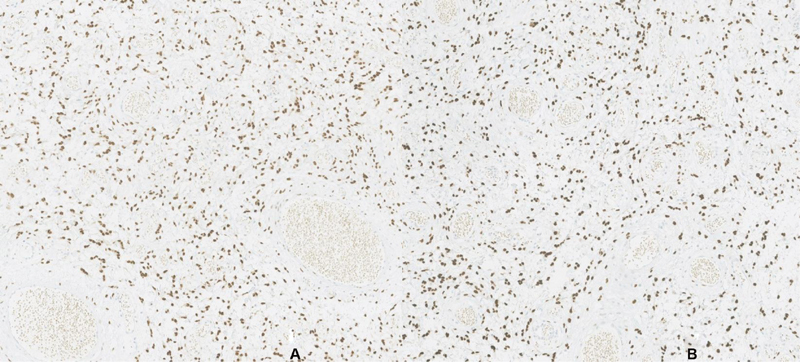
Immunohistochemistry: Estrogen (**A**) and progesterone (**B**) receptors expression.

The histopathological exam revealed a 24-g nodule with 5.5 cm, a smooth external surface, and an elastic, whitish and myxoid-appearing tissue. Microscopically, the lesion consisted of short spindle cells, accompanied by small collagen bundles, a prominent capillary vascular network, and multiple scattered mast cells (Figs. 1 and 2). There was no necrosis, cytological atypia, or identifiable mitosis. The immunohistochemical study showed diffuse tumor cell staining for estrogen and progesterone receptors (Fig. 3), and no staining for S100 protein, desmin, smooth muscle actin (SMA) or CD34. Less than 5% of the tumor cell population stained positive to Ki67. Scattered mast cells were highlighted by CD117. The vascular network was highlighted by CD34 and SMA.

The patient had a full and uneventful recovery and remains asymptomatic, with no evidence of local recurrence 10 months after the procedure.

## Discussion

Mesenchymal tumors in the vulvovaginal region are relatively rare, and they can be either non-specific tumors and have a more generalized distribution, such as those arising from smooth muscle (e.g., leiomyoma), vessels (e.g., hemangioma), or neural cells (e.g., granular cell tumors, schwannoma, neurofibroma), or they can have a predisposition to occur in the vulvovaginal region, such as aggressive angiomyxoma, angiomyofibroblastoma, or CA.^4^ ^6^

Given such tumoral diversity at this site, differential diagnosis of vulvar masses can be challenging. Most of these lesions present as nonspecific lesions, with similar characteristics, which might lead to a misdiagnosis that may convey a distinct prognosis.

Cellular angiofibroma is a rare benign tumor that originates from the mesenchymal cells of the blood vessels in superficial soft tissue.^2^ It occurs in middle-aged women, and its characteristic features are small masses (< 3 cm) with well circumscribed margins, often described as painless.

Histologically, these lesions are characterized by three components: spindle cells that form small fascicles surrounded by collagen bundles, numerous and prominent blood vessels, sometimes with hyalinized wall, and adipose tissue between fusiform cells.^1^ ^2^ ^3^ ^4^ ^6^ It shows immunostaining for vimentin, estrogen and progesterone receptors, smooth muscle actin and, in 60% of cases, also for CD34. There is no staining for S100 protein, desmin, keratins or epithelial membrane antigen (EMA).^1^ ^2^ ^3^ ^4^ ^6^ ^7^ The significance of estrogen and progesterone receptors expression is still uncertain. Since these receptors are normally expressed in the lower female genital tract mesenchymal cells, their presence in CA might be only a reflection of its cells origin.^4^ ^6^ ^7^ But, alternatively, some authors suggest that the expression of estrogen and progesterone receptors may play a role in the pathogenesis of the tumor.^4^ ^6^ ^7^

Although CA arises more frequently in the vulvovaginal region, there are some cases described in extrapelvic sites. In a review article by Mandato et al (2015)^4^ including 74 published cases of CA in women, most of the lesions were described as superficially located, especially at the vulvar site.^3^ Nonetheless, six cases presented lesions in extrapelvic locations, such as the left hip, lateral knee, chest wall, left axilla and breast, and left hypochondrium.

Cellular angiofibroma is commonly mistaken for a Bartholin gland, labial or submucosal cyst. Its distinction from other lesions is not only morphologic but also based on the immunohistochemical features. Establishing the diagnosis is of utmost importance because different lesions imply different prognosis and treatment.

The present patient had a painless vulvar mass, that was misdiagnosed as a Bartholin cyst, which is reported in some other published cases.^6^ ^8^ It was a painless lesion in a middle-age woman but larger than a typical CA, which are usually reported as small tumors.

Surgical excision of the lesion with tumor-free margins seems to be the adequate management to these lesions.^9^ It allows not only to treat the patient but also to achieve a correct diagnosis. The potential recurrence risk of these lesions is low, as there is only one case of recurrence reported in the literature, and no known malignant transformation.^6^ ^8^ ^10^ The lack of long-term data also limits the establishment of adequate surveillance for these patients, so a similar approach to other benign gynecological lesions may be undertaken.

## Conclusion

Cellular angiofibroma is a rare vulvar tumor that should be considered in the differential diagnosis of vulvar masses. The potential recurrence risk of these lesions is low, but its prognosis remains unclear as data on long-term follow-up is not presently available.
